# Correlation between different levels of Her-2 protein expression and the efficacy of neoadjuvant therapy in Her-2 positive breast cancer

**DOI:** 10.3389/fonc.2025.1673234

**Published:** 2025-11-06

**Authors:** Wen-hui Wang, Hong-bo Li, Zhong-yuan Cui, Yan-dong Li, Zhi-yong Wang

**Affiliations:** 1 Breast Surgery, BeiHua University Affiliated Hospital, Jilin, Jilin, China; 2 Breast and Thyroid Surgery, Jinhua People’s Hospital, Jinhua, Zhejiang, China

**Keywords:** Her-2 protein, breast cancer, trastuzumab, pertuzumab, pCR

## Abstract

**Introduction:**

The purpose of this study was to explore the correlation between the difference of Her-2 protein expression level in Her-2 positive breast cancer and the efficacy of neoadjuvant chemotherapy, so as to determine the best population for neoadjuvant treatment of Her-2 positive breast cancer.

**Methods:**

This study enrolled 161 Her-2 positive breast cancer patients who received trastuzumab plus pertuzumab therapy between January 2020 and January 2024. Statistical analyses were employed to evaluate associations between Her-2 protein expression levels and clinicopathological characteristics, as well as relationships between pathological complete response (pCR) and these features. Logistic regression was used to assess correlations between Her-2 expression levels and clinicopathological factors, and to analyze pCR predictors.

**Result:**

Among the 161 patients receiving neoadjuvant trastuzumab-pertuzumab therapy, 81 achieved pCR (50.31%), while 80 did not (49.69%). In the Her-2 (3+) subgroup (n=134), 58.21% (78/134) attained pCR versus 41.79% (56/134) with non-pCR. For Her-2 (2+)/FISH(+) patients (n=27), only 11.11% (3/27) achieved pCR, contrasting with 88.89% (24/27) showing non-pCR. Univariate analysis identified hormone receptor status, tumor size, and Her-2 expression level as pCR influencers. However, multivariate logistic regression confirmed Her-2 expression as the sole independent predictor: Her-2 (3+) patients had significantly higher pCR rates than Her-2 (2+)/FISH(+) cases (p=0.005; OR: 0.170 [95% CI: 0.045–0.639]).

**Conclusion:**

Her-2 (3+) expression correlates with superior pCR rates, underscoring the need for further research to refine patient selection for optimized targeted therapy benefits.

## 1 Introduction

Breast cancer, with an estimated 2.3089 million new cases and 665,700 deaths worldwide in 2022, remains the most prevalent cancer among women globally, exerting a profound impact on their lives and health (1). Breast cancer can be categorized into four molecular subtypes based on the overexpression of Her-2 protein and hormone receptors. The advent of trastuzumab in clinical practice has significantly improved the survival rates of Her-2 positive breast cancer patients, who were previously among those with the poorest prognosis. Neoadjuvant therapy not only increases the likelihood of breast and axillary conserving surgery but also plays a crucial role in identifying patients who have not achieved pathological complete response (pCR), thereby enabling the provision of intensified postoperative treatment to enhance survival times. Patients who attain pCR have been shown to have a more favorable prognosis compared to those who do not (2). The cornerstone of targeted therapy lies in identifying precise therapeutic targets. However, in clinical practice, despite the efficacy of targeted therapies for Her-2 positive breast cancer, it remains unclear which factors influence pCR following neoadjuvant treatment. Moreover, both Her-2 immunohistochemical (IHC) (3+) and Her-2 (2+)/FISH (+) patients are classified as Her-2 positive breast cancer; it is yet to be determined whether different levels of Her-2 protein expression will yield the same prognosis under the same dual-target neoadjuvant therapy regimen.

## 2 Materials and methods

### 2.1 Patients

Inclusion Criteria:

1. Patients provided pathological and immunohistochemical (IHC) results through core needle biopsy at admission, with pathological diagnoses of invasive breast cancer and IHC results indicating Her-2 (3+) or Her-2 (2+)/FISH (+), ER, PR, Ki-67;
2. Patients completed 6–8 cycles of neoadjuvant therapy, including trastuzumab and pertuzumab, in accordance with the breast cancer diagnosis and treatment guidelines;
3. Post-neoadjuvant therapy, patients underwent surgical treatment, including breast-conserving radical surgery, modified radical surgery, breast reconstruction, sentinel lymph node biopsy, and axillary lymph node dissection. Comprehensive pathological and immunohistochemical results, including but not limited to Her-2, ER, PR, and Ki-67, were obtained post-surgery, and follow-up treatment was completed as per guidelines:
1. Patients who declined biopsy or had incomplete clinical and pathological data upon admission;
2. Patients unable to complete the neoadjuvant therapy cycle, refused surgery, or declined follow-up treatment post-surgery;
3. Non Her-2 positive breast cancer patients or those with postoperative immunohistochemical results inconsistent with preoperative core needle biopsy pathological results;
4. Male breast cancer patients; inflammatory breast cancer patients; pregnant women or those with significant comorbidities intolerant to chemotherapy, targeted therapy, or surgery;

Selection of Neoadjuvant Therapy Population and Specific Plan:

In accordance with the 2020–2024 Chinese Society of Clinical Oncology (CSCO) guidelines for breast cancer diagnosis and treatment:

Neoadjuvant therapy population: patients with tumors >5cm; positive axillary lymph nodes; triple-negative breast cancer, Her-2 positive breast cancer; those requiring breast and axillary conserving surgery;

Specific plan and dosage:

TCbHP consists of 6 cycles (docetaxel 75mg/m^2 iv. D1, carboplatin AUC = 6 iv. D1, trastuzumab initial dose 8mg/kg followed by 6mg/kg, pertuzumab initial dose 840mg followed by 420mg); AC-THP consists of 8 cycles (doxorubicin 75-100mg/m^2 iv. D1, cyclophosphamide 600mg/m^2 iv. D1, docetaxel 80-100mg/m^2 iv. D8, trastuzumab initial dose 8mg/kg followed by 6mg/kg, pertuzumab initial dose 840mg followed by 420mg).

Pathological and imaging result evaluation:

Pathological results were evaluated based on the 2018 American Society of Clinical Oncology (ASCO)/College of American Pathologists (CAP) criteria: ER and PR positivity criteria were nuclear staining >1%; Her-2 (3+) definition: >10% of invasive cancer cells showing strong, complete, and uniform cell membrane staining; Her-2 (2+) definition: >10% of invasive cancer cells showing moderate to complete cell membrane staining with moderate intensity, or ≤10% of invasive cancer cells showing strong, complete, and uniform cell membrane staining. Her-2 (2+) requires further original hybridization testing (FISH or CISH); Ki-67 is considered high at a threshold of 20%; pCR definition: No infiltrating cancer cells detected in the breast and axillary lymph nodes. Imaging results were evaluated according to RECIST 1.1 criteria: CR: complete remission; PR: partial response; SD: stable disease; PD: progressive disease.

### 2.2 Statistical analysis

All data analyses were performed using SPSS 25.0 (SPSS.). Chi-square tests/Fisher’s exact tests were applied to analyze the correlation between the clinical and pathological outcomes of the Her-2 (3+) and Her-2 (2+)/FISH (+) patient groups; logistic regression was used to analyze the correlation between clinicopathological features and pCR. A p-value of <0.05 was considered statistically significant.

### 2.3 Ethical approval and consent to participate

This study was conducted in accordance with the principles of the Declaration of Helsinki and was approved by the Independent Ethics Committee of the Affiliated Hospital of Beihua University. Due to the retrospective design of this study, the Ethics Committee of the Affiliated Hospital of Beihua University granted a waiver of informed consent.

## 3 Result

### 3.1 Patient characteristics

Between January 2020 and January 2024, a cohort of 161 female patients with Her-2 positive breast cancer was enrolled from four regional breast diagnosis and treatment centers. Of these, 81 patients achieved pathological complete response (pCR) following neoadjuvant therapy, representing 50.31% of the study population, while 80 patients did not achieve pCR, accounting for 49.69%. Among the patients, 134 had Her-2 (3+) expression, with 78 achieving pCR (58.21%) and 56 not achieving pCR (41.79%). For the 27 patients with Her-2 (2+)/FISH (+), 3 achieved pCR (11.11%), and 24 did not (88.89%).

Univariate analysis revealed that estrogen receptor (ER) and progesterone receptor (PR) status were factors influencing HER2 protein expression levels. Multivariate logistic regression analysis identified PR status as the sole significant predictor of HER2 protein expression (**Table 1**, **2**).

**Table 1:** Her-2 protein expression level and clinical pathological characteristics.

Characteristic	N	Her-2(2+)/FISH(+)	Her-2(3+)	χ^2^/Z	*p*
year				0.012	0.914
<50	76	13	63		
≥50	85	14	71		
menopause				0.546	0.460
yes	79	15	64		
no	82	12	70		
TNM				1.630	0.653
T1	23	3	20		
T2	115	21	94		
T3	17	3	14		
T4	6	0	6		
lymph node				0.132	0.717
positive	127	22	105		
negative	34	5	29		
ER					**0.001**
positive	90	25	65		
negative	71	2	69		
PR					**0.001**
positive	73	23	50		
negative	88	4	84		
Ki-67				0.041	0.839
<30%	51	9	42		
≥30%	110	18	92		
scheme				1.606	0.205
TCbHP	102	20	82		
AC-THP	59	7	52		

Bold values indicate statistical significance (p < 0.05).

**Table 2:** Multivariate logistic regression analysis of factors associated with high Her-2 protein expression (IHC 3+).

Variables	Wald	OR	95%CI	*p*
ER	3.222	5.127	0.861-30.542	0.073
PR	4.658	4.658	1.1521-8.839	**0.031**

ER, estrogen receptor; PR, progesterone receptor; TCbHP, Paclitaxel, carboplatin, trastuzumab, Pertuzumab; AC, Adriamycin, cyclophosphamide. Bold values indicate statistical significance (p < 0.05).

According to univariate analysis, the relevant factors affecting pCR include TNM stage, ER, PR status, and Her-2 protein expression level. After conducting multivariate logistic regression analysis, Her-2 protein expression was identified as the sole influencing factor affecting pCR. In multiple regression analysis, the pCR of patients with Her-2 (3+) protein expression was significantly higher than that of Her-2 (2+)/FISH (+) patients (p=0.005 OR: 0.170 [95% CI: 0.045-0.639]). (**Table 3**, **4**).

**Table 3:** Related factors affecting pCR.

Characteristic	N	pCR	non-pCR	χ^2^/Z	*p*
year				0.058	0.809
<50	76	39	37		
≥50	85	42	43		
menopause				3.889	0.049
yes	82	35	47		
no	79	46	33		
TNM					**0.002**
T1	23	16	7		
T2	115	53	62		
T3	17	8	9		
T4	6	4	2		
lymph node				0.119	0.730
positive	127	63	64		
negative	34	18	16		
ER				23.530	**0.001**
positive	90	30	60		
negative	71	51	20		
PR				24.795	**0.001**
positive	73	21	52		
negative	88	60	28		
Ki-67				0.050	0.823
<30%	51	25	26		
≥30%	110	56	54		
scheme				1.869	0.172
TCbHP	102	48	54		
AC-THP	59	33	26		
Protein expression					**0.001**
3+	134	78	56		
2+	27	3	24		

Bold values indicate statistical significance (p < 0.05).

**Table 4:** Multivariate logistic regression analysis of pCR.

Variables	Wald	OR	95%CI	*p*
TNM	4.369			
T1	0.004	1.068	0.137-8.313	0.950
T2	1.127	0.371	0.059-2.316	0.288
T3	0.675	0.420	0.053-3.331	0.411
ER	2.863	2.256	0.875-5.816	0.095
PR	3.041	2.344	0.900-6.108	0.081
Her-2	6.873	0.170	0.045-0.639	**0.009**

Bold values indicate statistical significance (p < 0.05).

## 4 Discussion

Her-2 positive breast cancer, previously associated with poor outcomes, has shown markedly improved survival rates following the clinical adoption of trastuzumab (3). This monoclonal antibody binds the extracellular domain of Her-2, suppressing tyrosine kinase signaling and subsequent tumor growth. Combining pertuzumab with trastuzumab in adjuvant or neoadjuvant regimens has demonstrated superior pathological complete response (pCR) rates and five-year survival compared to single-agent therapy (4, 5). As a humanized monoclonal antibody targeting a different HER2 epitope, pertuzumab synergizes with trastuzumab to block homodimer and heterodimer formation—critical events in Her-2 mediated oncogenic signaling. Achieving pCR before surgery consistently correlates with favorable long-term outcomes in Her-2 positive breast cancer (2), motivating extensive research into pCR predictors for this subtype (6). Clinical and molecular evidence confirms that elevated Her-2 expression not only indicates aggressive disease but also underpins the therapeutic rationale for Her-2 targeted interventions. Following the 2018 ASCO/CAP Her-2 protein scoring guidelines, we observed that patients with Her-2 immunohistochemistry 3+ consistently achieved higher overall pCR rates. We analyzed pooled data from the Peripheral Breast Diagnosis and Treatment Center, enrolling 161 Her-2-positive breast cancer patients receiving trastuzumab and pertuzumab dual-target neoadjuvant therapy. Applying strict selection criteria yielded an overall pCR rate of 50.31%, consistent with NeoSphere, KRISTINE, and TRAIN-2 trial outcomes. Notably, Her-2 (3+) patients comprised 96.30% of the pCR subgroup, while Her-2 (2+)/FISH (+) cases represented only 3.7%. Subsequent statistical analysis confirmed Her-2 (3+) overexpression significantly predicted pCR compared to Her-2 (2+)/FISH (+) status (p=0.001), with an odds ratio of 0.17 in multivariate analysis. We conducted a PubMed literature review of “breast cancer, Her-2, neoadjuvant, pertuzumab” to contextualize these findings, excluding non-Her-2 positive and single-target therapy studies (**Table 5**). The CSBrS-026 trial (7), involving 2395 patients, similarly showed Her-2 (3+) cases achieving 94.25% pCR rates versus Her-2 (2+)/FISH (+), corroborating our results. Both our univariate analysis and this prior work identified hormone receptor status as another significant pCR predictor. Emerging evidence indicates that bidirectional crosstalk between ER and Her-2 signaling pathways may explain this interaction, where ER pathway modulation during targeted therapy for ER positive breast cancer could induce Her-2 treatment resistance through various mechanisms (8, 9). While our multivariable regression analysis showed no statistically significant associations, a consistent trend emerged. The relatively small cohort in our study likely limited statistical power, potentially accounting for discrepancies with the CSBrS-026 trial results. Multiple studies have established Her-2 (3+) protein expression as a robust predictor of pCR in Her-2 positive breast cancer (10–17). Lin et al.’s work similarly reported no significant association between hormone receptor status and pCR in Her-2 expressing tumors, corroborating our observations (13). Their analysis attributed these negative findings to limited sample size, highlighting the importance of future hormone receptor investigations. Our data revealed higher Her-2 overexpression rates among hormone receptor-negative patients, consistent with clinical experience. Although some reports found no association between Her-2 expression levels and pCR (18–20), this potential relationship warrants additional rigorous evaluation through larger-scale studies.

**Table 5:** Study on trastuzumab+pertuzumab as NAT for Her-2 Positive breast cancer.

Author	Year	Country	Her-2	pCR	non-pCR	*p*
Hongyu Xiang (7)	2023	China	3+	1197	843	0.001
			2+	73	282	
Hang Lu (10)	2023	China	3+	86	56	0.001
			2+	3	14	
Dat Le (11)	2022	USA	3+	62	64	0.001
			2+	1	26	
Dong-Mei Peng (12)	2024	China	3+	102	55	0.003
			2+	22	15	
Binwei Lin (13)	2023	China	3+	35	28	0.013
			2+	4	14	
Wei Chen (14)	2023	China	3+	119	69	0.015
			2+	15	21	
Adrienne G Waks (15)	2023	USA	3+	45	23	0.039
			2+	3	7	
Xiangmin Ma (16)	2022	China	3+	191	75	0.001
			2+	5	31	
Laura Spring (17)	2019	USA	NR	NR	NR	0.03
Fanfan Li (18)	2022	China	3+	30	10	0.523
			2+	11	6	
Yi Xiao (19)	2022	China	3+	97	42	0.184
			2+	10	10	
Katalin Boér (20)	2021	Hungary	3+	38	29	0.241
			2+	6	9	

The background of this trial stems from clinical observations that Her-2 (2+)/FISH (+) patients exhibit low pCR rates. Through retrospective analysis of extensive clinical data, we found that dual-target therapy yields suboptimal outcomes in Her-2 (2+)/FISH (+)patients, a conclusion corroborated by subgroup analyses in multiple studies. Identifying this treatment-resistant population underscores the need for clinicians to prioritize Her-2 (2+)/FISH (+) cases. Delving deeper into the mechanisms behind poor targeted therapy responses in Her-2 (2+)/FISH (+)patients, we noted that current Her-2 positivity criteria were established based on historical clinical trial cohorts. Given the recognized inefficacy in Her-2 (2+)/FISH (+) subgroups, the evolving landscape of anti-Her-2 therapies—exemplified by DS-8201’s redefinition of low Her-2 expression prompts reconsideration of optimal treatment plans for this population (21). Novel agents with superior efficacy may await discovery. Further investigation revealed potential beneficiary subgroups among Her-2 (2+)/FISH (+) patients. Limited by small sample sizes, we propose refining classifications via Her-2 genetic testing. As early as 2022, Eric M. Lander (22) demonstrated that HER-2/CEP17 FISH ratios influence pCR rates, a finding echoed by Chinese researchers (23) showing Her-2/CEP17 ratios ≥6.0 correlate with higher pCR. This suggests Her-2-positive breast cancer warrants finer stratification: Her-2 (3+) patients may benefit from dual-target or Tyrosine Kinase Inhibitors (TKIs) therapy, while Her-2 (2+)/FISH (+) patients could be treated based on Her-2/CEP17 ratios. Intriguingly, TKIs like pyrotinib combined with trastuzumab have shown promising pCR in Chinese studies, though conclusive evidence remains pending. These clinical insights continually inspire progress, ultimately advancing patient care.

This study stands as the sole retrospective analysis focusing on the significance of Her-2 (3+) protein as a predictor of pCR in Her-2 positive breast cancer treated with dual-target neoadjuvant therapy. However, it is not devoid of limitations: (1) The sample size was insufficient, primarily due to the economic costs of dual-target therapy. It is recommended that future studies enroll at least 200 patients to address pertinent questions;(2) For Her-2 (3+) patients with high dual-target pCR and favorable outcomes, should the focus be on targeted therapy or chemotherapy for Her-2 (2+)/FISH (+) patients, and how should drug therapy be adjusted? (3) While our study used conventional IHC/FISH criteria, we recognize that Her-2 biology is more continuous than binary. Two factors may explain the poor response in Her-2 (2+)/FISH+ patients: Intratumoral heterogeneity in HER2 expression and amplification, particularly in IHC 2+ tumors, may lead to sampling bias and therapy resistance (24).The emerging concept of Her-2-low disease (IHC 1+ or 2+/FISH-negative) and its response to novel ADCs (T-Dxd) suggest that Her-2 (2+)/FISH+ tumors may reside in a biological ‘gray zone’ (21). Their behavior may align more with Her-2-low biology, suggesting potential benefit from ADCs rather than traditional dual blockade; (4) Some studies suggest a correlation between hormone receptor status, androgen receptor, CK5/6, and Her-2 protein expression levels, which requires additional research data for validation. (5) Although pCR is a validated surrogate endpoint for long-term survival in HER2-positive breast cancer treated with neoadjuvant anti-HER2 therapy (2), direct assessment of event-free survival (EFS) and overall survival (OS) will provide more definitive evidence. We are actively collecting long-term follow-up data for future analysis. (6) This study was conducted within a single healthcare network in Jilin Province, China. While we adhered to national guidelines and standardized protocols, the results may reflect institutional preferences in treatment selection and pathological assessment. The relative homogeneity of our patient population may limit generalizability to other geographic or ethnic groups. Future multi-center studies incorporating central pathology review are warranted to validate our findings.

## 5 Conclusion

The combination of trastuzumab and pertuzumab represents a standard neoadjuvant regimen for Her-2 positive breast cancer, particularly in patients with Her-2 3+ overexpression. and the prognosis of patients who reach pCR will be better. Patients with Her-2 protein (3+) will definitely have higher pCR. Optimizing ADC deployment and chemotherapy regimens remains challenging. We are prospectively tracking this cohort to analyze EFS and OS, which will be reported in a subsequent study.

## Data Availability

The original contributions presented in the study are included in the article/- **Supplementary Material**. Further inquiries can be directed to the corresponding author.
